# Two new bamboo-feeding species of the genus *Kirbyana* Distant, 1906 from China (Hemiptera, Fulgoromorpha, Cixiidae)

**DOI:** 10.3897/zookeys.1037.64653

**Published:** 2021-05-11

**Authors:** Yan Zhi, Lin Yang, Xiang-Sheng Chen

**Affiliations:** 1 Laboratory Animal Center, Guizhou Medical University, Guiyang, Guizhou, 550025, China; 2 Institute of Entomology, Guizhou University, Guiyang, Guizhou, 550025, China; 3 The Provincial Special Key Laboratory for Development and Utilization of Insect Resources of Guizhou, Guizhou University, Guiyang, Guizhou, 550025, China

**Keywords:** Auchenorrhyncha, Cixiidae, Fulgoromorpha, Oriental region, planthopper, taxonomy

## Abstract

Two new bamboo-feeding species of the cixiid planthopper genus *Kirbyana* Distant, 1906 (Hemiptera: Fulgoromorpha: Cixiidae: Eucarpiini), *K. aspina* Zhi & Chen, **sp. nov.** and *K. furcata* Zhi & Chen, **sp. nov.**, are described and illustrated from southern China. A key to all known species and a map of their geographic distributions are provided.

## Introduction

The planthopper genus *Kirbyana* was established by Distant (1906), with *Kirbya pagana* Melichar, 1903 as the type species. This genus belongs to the tribe Eucarpiini of subfamily Cixiinae (Hemiptera: Cixiidae) (Holzinger et al. 2002). Previously, eight species and one subspecies in this genus have been recorded: *Kirbyana australis* (Muir, 1913), *K deusta* (Distant, 1911), *K deventeri* (Kirkaldy, 1907), *K javana* Muir, 1913, *K lini* Tsaur & Hsu, 2003, *K pacifica* Emeljanov & Hayashi, 2007, *K. pagana* (Melichar, 1903), *K pratti* Muir, 1913 and *K pratti thyas* Fennah, 1978 (Bourgoin 2021).

Recent study of some Chinese specimens has found two new bamboo-feeding species, *K. aspina* Zhi & Chen, sp. nov. and *K. furcata* Zhi & Chen, sp. nov., which are described here. So far, including the two new species, the genus currently now counts for ten valid species, all distributed in the Australian and Oriental regions (Australia, China, India, Indonesia, Japan, Malaysia, Sri Lanka and Vietnam).

## Materials and methods

The morphological terminology follows Bourgoin (1987) for male genitalia, Bourgoin et al. (2015) for wing venation and Bourgoin (1993) for female genitalia. Body length was measured from apex of vertex to tip of forewing; vertex length represented the median length of the vertex (from the apical transverse carina to the tip of basal emargination). Fuchsin staining was used to highlight female genitalia structures studied. External morphology and drawings were done with the aid of a Leica MZ 12.5 stereomicroscope. Photographs were taken with a KEYENCE VHX-6000 system. Illustrations were scanned with a CanoScan LiDE 200 and imported into Adobe Photoshop 7.0 for labeling and plate composition. The dissected male and female genitalia are preserved in glycerin in small plastic tubes pinned together with the specimens.

The type specimens are deposited in the Institute of Entomology, Guizhou University, Guiyang, Guizhou Province, China (**GUGC**).

## Taxonomy

### Genus Kirbyana

Taxon classificationAnimaliaHemipteraCixiidae

Distant, 1906

BF32FA70-E30F-52EB-9BEE-16F47A65C7B6

Kirbya Melichar, 1903: 37, preoccupied by Kirbya Robineau-Desvoidy, 1830 (Diptera).
Kirbyana Distant, 1906a: 262, nom. nov. for Kirbya Melichar, 1903.
Kirbyella Kirkaldy, 1906: 248, synonymised by Distant 1906b: 274.
Saccharias Kirkaldy, 1907: 125, synonymised by Fennah 1980: 239.
Commolenda Distant, 1911: 741, synonymised by Fennah 1978: 211.

#### Type species.

*Kirbya pagana* Melichar, 1903 (original designation by Distant).

#### Diagnosis.

***Head*.** Head including eyes slightly narrower than pronotum. Vertex in profile horizontal, in same line as thorax, meeting frons abruptly nearly at 90°; subapical carina absent. Frons somewhat longer than broad or as long as wide; median carina present; anterior margin angularly emarginate or transverse; position of maximum width of frons more or less at the level of antennae. Median ocellus absent. Subapical segment of rostrum 2.5 times longer than apical segment.

***Thorax*.** Pronotum very narrow, scarcely half as long as vertex in midline. Mesonotum nearly twice as long as pronotum and vertex together. Forewing with the eucarpian basal concavity of the anterior margin of the forewings, also slightly concave at node level. Hindwing with simple radius (R). Hind tibia lacking lateral spines. Metatibiotarsal formula: 6/8‒11/9‒11.

***Male genitalia*.** Pygofer symmetrical and prolonged with symmetrical lateral lobes in lateral view. Medioventral process thumb-like in lateral view. Anal segment short and stout. Gonostyli relatively small and symmetrical. Aedeagus slender and endosoma of aedeagus with spinose processes.

***Female genitalia*.** Ovipositor elongate, orthopteroid and slightly curved upwards; anal segment square or rectangular in dorsal view; 9^th^ tergite without wax plate. Posterior vagina with sclerites.

#### Distribution.

Australian and Oriental regions.

##### Checklist and distributions of species of *Kirbyana* Distant

*K. aspina* Zhi & Chen, sp. nov.; China (Hunan).

*K. australis* (Muir, 1913); Australia (Northern Territory; Queensland).

*K deusta* (Distant, 1911); Central India.

*K deventeri* (Kirkaldy, 1907); Indonesia (Java).

*K. furcata* Zhi & Chen, sp. nov.; China (Guangxi, Yunnan).

*K javana* Muir, 1913; Indonesia (Java).

*K lini* Tsaur & Hsu, 2003; China (Taiwan).

*K pacifica* Emeljanov & Hayashi, 2007; China (Taiwan), Japan (Ryukyu Islands).

*K. pagana* (Melichar, 1903); Sri Lanka (Peradeniya).

*K pratti* Muir, 1913; Malaysia (Parit Buntar).

*K pratti thyas* Fennah, 1978; Vietnam (Cuc-phuong Province, Ninh Binh).

##### Key to the species (males) of *Kirbyana* Distant

**Table:** 

1	Anterior margin of vertex transverse	**2**
–	Anterior margin of vertex angularly excavated or incised at middle	**7**
2	Forewing with numerous small fuscous spots on basal half and several waved fuscous lines on apical half	***K deusta* (Distant, 1911)**
–	Forewing not so marked	**3**
3	Forewing with a transverse veinlet from M to CuA near Cu fork	**4**
–	Forewing without transverse veinlet from M to CuA near Cu fork	**5**
4	Forewing without transverse veinlet from MP3+4 to CuA1 (Löcker et al. 2010: fig. 6C)	***K. australis* (Muir, 1913)**
–	Forewing with two transverse veinlets from MP3+4 to CuA1 (Kirkaldy 1907: fig. 1)	***K deventeri* (Kirkaldy, 1907)**
5	Ventral margin of periandrium without spinous processes near base (Tsaur and Hsu 2003: fig. 2D)	***K lini* Tsaur & Hsu, 2003**
–	Ventral margin of periandrium with two spinous processes near base	**6**
6	One of the two basal processes of ventral margin of periandrium bifurcate (Fig. 5I, J, L)	***K. furcata* sp. nov.**
–	Both basal processes of ventral margin of periandrium unbifurcated (Fig. 2I, J, L)	***K. aspina* sp. nov.**
7	All tubercles of longitudinal veins in forewings colourless	***K javana* Muir, 1913**
–	Some tubercles of longitudinal veins in forewings dark	**8**
8	Forewing with MP joining CuA with crossvein M_3+4_-CuA (Emeljanov and Hayashi 2007: fig. 25)	***K pacifica* Emeljanov & Hayashi, 2007**
–	Forewing with MP joining CuA directly without crossvein M_3+4_-CuA	**9**
9	Forewing with a series of black spots on the claval vein and the inner bifurcating veins; mesonotum with a small dark spot near base of each lateral carina	***K. pagana* (Melichar, 1903)**
–	Forewing not so marked; mesonotum without a spot on each side near base of lateral carina	***K pratti* Muir, 1913**

### Kirbyana aspina

Taxon classificationAnimaliaHemipteraCixiidae

Zhi & Chen sp. nov.

DE2FE23F-CD21-5C4A-BCCC-6A41F7A299E2

http://zoobank.org/9FF2E020-333D-4CCD-AFED-FA322162E171

Figures 1 , 2 , 3

#### Type material.

***Holotype*:** ♂, China: Hunan Province, Wugang City, Yunshan National Forest Park (26°40'N, 110°37'E), 5 June 2011, leg. Xiang-Sheng Chen; ***Paratypes***: 8♂♂7♀♀, same data as holotype.

#### Description.

Body length: male 5.6‒6.1 mm (*N* = 9), female 5.9‒6.5 mm (*N* = 7).

**Figure 1.**
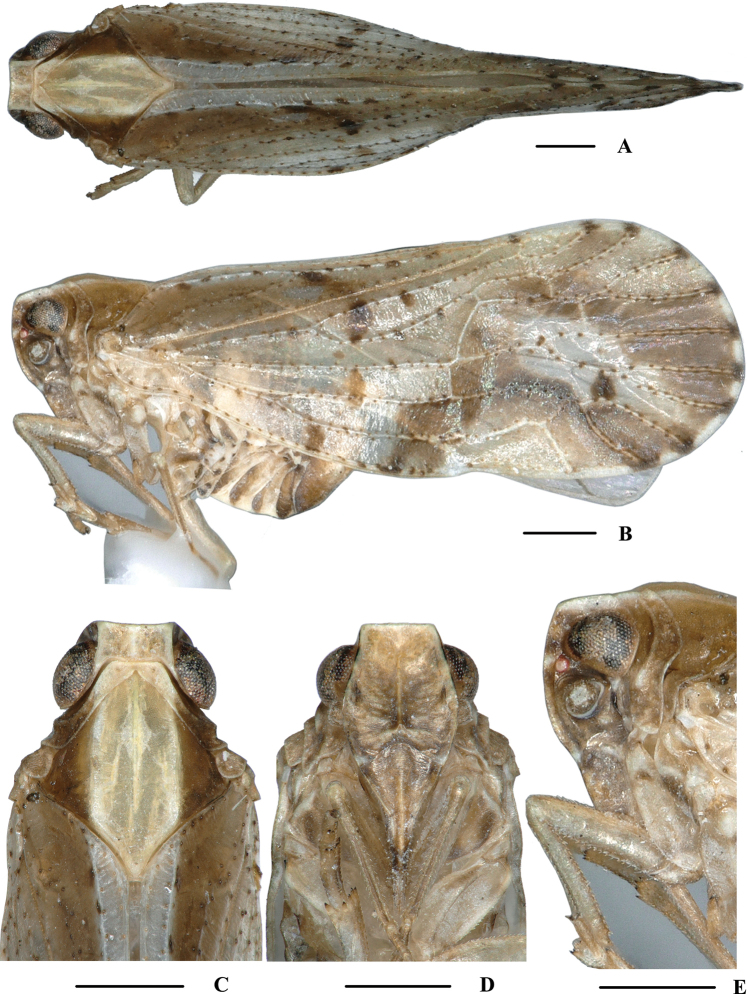
*Kirbyana aspina* sp. nov., male **A** habitus, dorsal view **B** habitus, lateral view **C** head and thorax, dorsal view **D** face, ventral view **E** head, lateral view. Scale bars: 0.5 mm.

***Coloration*.** General color light brown (Figs 1A–E, 3J, K). Eyes blackish brown, ocelli light yellow, semitransparent. Vertex generally yellowish white. Face generally dark brown; rostrum light brown. Pronotum with discal areas and mesonotum with area between lateral carinae yellowish white, lateral areas brown. Forewing light brown, semi-translucent. Stigma light brown. The basal half dotted with small dark brown spots and distal half with two large dark brown patches; small dark brown spots on the ends of longitudinal veins. Hind tibiae and abdominal sternites yellowish brown.

**Figure 2.**
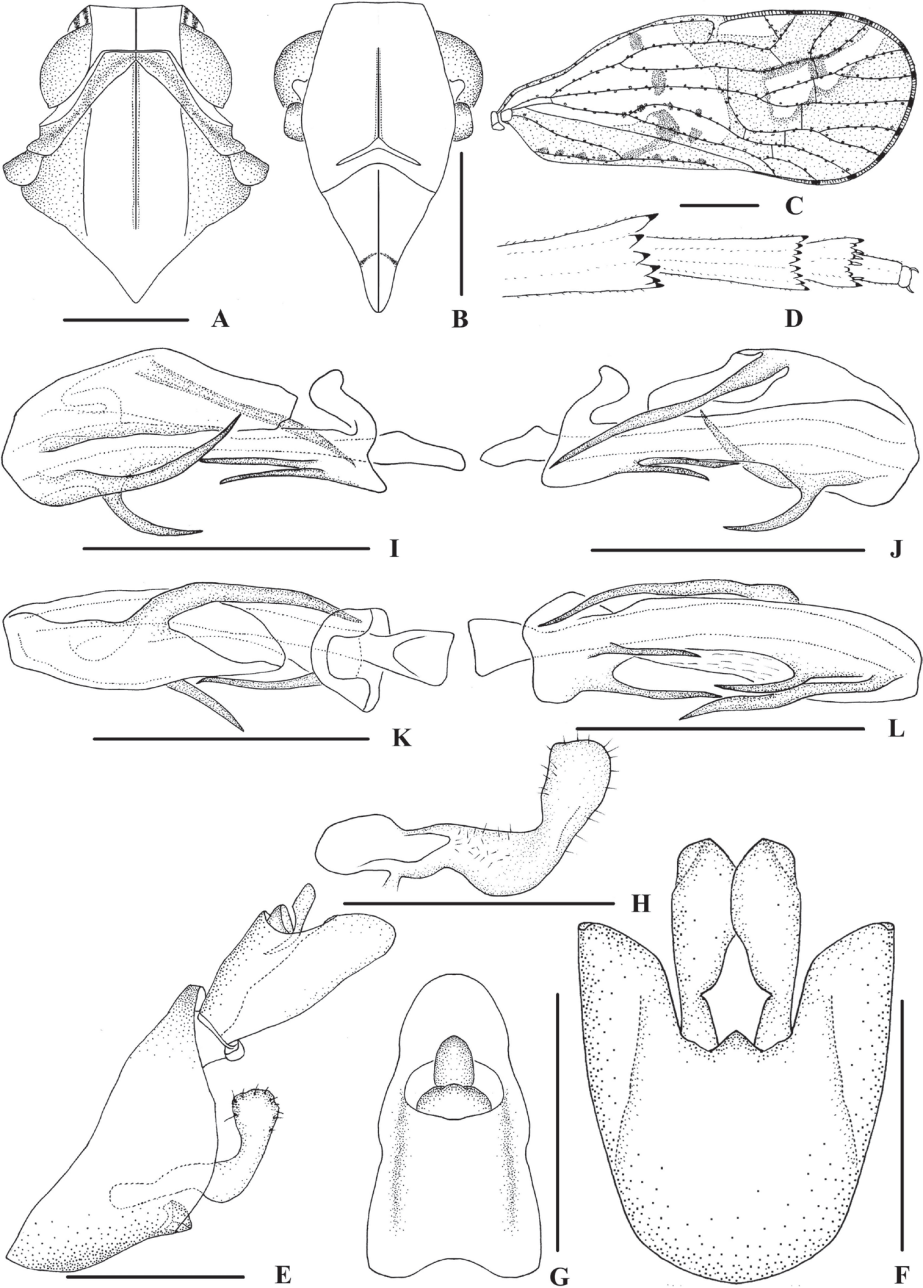
*Kirbyana aspina* sp. nov., male **A** head and thorax, dorsal view **B** face, ventral view **C** forewing **D** apex of left hind leg, ventral view **E** genitalia, lateral view **F** pygofer and gonostyli, ventral view **G** anal segment, dorsal view **H** gonostyli, inner lateral view **I** aedeagus, right side **J** aedeagus, left side **K** aedeagus, dorsal view **L** aedeagus, ventral view. Scale bars: 0.5 mm (**A, B, E–L**); 1.0 mm (**C**).

***Head and thorax*.** Vertex (Figs 1C, 2A) broad, 2.2 times wider than long; anterior margin truncated, posterior margin arched and recessed. Frons (Figs 1D, 2B) widest at the level of antennae, as long as wide; frontoclypeal suture nearly concave into an arch; middle carina with basal half absent; lateral carinae distinct and slight elevated. Rostrum distinctly surpassing hind coxae, subapical segment 2.5 times longer than apical segment. Pronotum (Figs 1C, 2A) 2.4 times longer than vertex; median carina indistinct, posterior margin nearly at right angle. Mesonotum 1.7 times longer than pronotum and vertex combined. Forewing (Fig. 2C) 2.4 times longer than wide, with 10 apical and 6 subapical cells; fork Sc+RP slightly basad of fork CuA_1_+CuA_2_, first crossvein r-m at same level of fork MP, RP two branches, MP with five terminals: MP_11_, MP_12_, MP_2_, MP_3_ and MP_4_, fork MP_1_+MP_2_ basad of fork MP_3_+MP_4_. Metatibiotarsal formula: 6/8–9/9, second segment of hind tarsus with four platellae (Fig. 2D).

***Male genitalia*.** Pygofer (Fig. 2E, F) symmetrical, dorsal margin concave and U-shaped, slightly widened towards apex in ventral view; in lateral view, lateral lobes extended in an arc caudally, medioventral process triangular in ventral view. Anal segment (Fig. 2E, G) broad, dorsal margin almost straight, apical half of ventral margin convex, apical lobes round in lateral view; 1.7 times longer than wide in dorsal view; anal style strap-shaped, not beyond anal segment. Gonostyli (Fig. 2E, F, H) symmetrical in ventral view; in inner lateral view, base of ventral margin concave, dorsal margin bending inwards at a nearly right angle in the middle, apical part extended, apical margin round. Aedeagus (Fig. 2I–L) with total of five processes. On right side, apex of periandrium with a long spinous process, sinuous, apex directed right-dorsocephalically; basal 1/4 of ventral margin with two short spinous processes, the longer one straight, directed caudally, the shorter one slightly curved and apex directed ventrocaudally; apical 1/3 of ventral margin with a curved spinous process, apex directed apically. Endosoma moderately sclerotised, relatively short, generally curved dorsally. The left dorsal margin with a long spinous process, slightly curved, and apex directed ventrocephalically.

**Figure 3.**
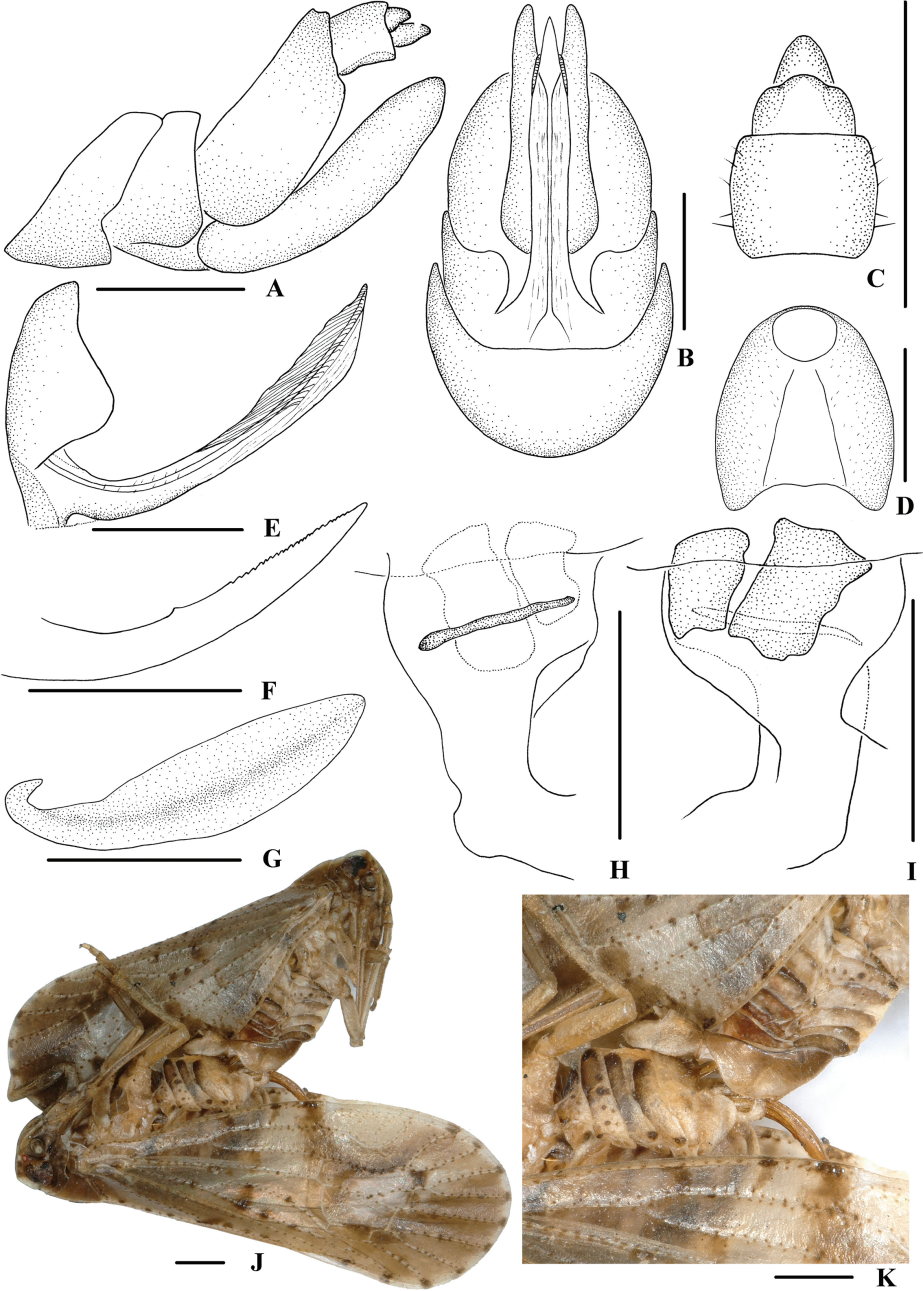
*Kirbyana aspina* sp. nov., **A–I** female **A** genitalia, lateral view **B** genitalia, ventral view **C** anal segment, dorsal view **D** tergite IX, caudal view **E** gonapophysis VIII and gonocoxa VIII, ventral view **F** gonapophysis IX, lateral view **G** gonoplac, lateral view **H** posterior vagina, dorsal view **I** posterior vagina, ventral view **J, K** mating. Scale bars: 0.5 mm.

***Female genitalia*.** Posterior margin of pregenital sternite concave. Tergite IX (Fig. 3A, D) moderately sclerotised, with length almost equal to width in caudal view. Anal tube (Fig. 3A, C) short, nearly rectangular, slightly widened towards apex, 1.5 times longer than wide in dorsal view; dorsal and ventral margins nearly straight in lateral view, anal styles strap-shaped. Gonapophysis VIII (Fig. 3E) elongate, and slightly curved upwards. Gonapophysis IX (Fig. 3F), distance ratio between middle tooth to apex and length of denticulate portion is 1.50. Gonoplac (Fig. 3G) rod-like, 3.7 times longer than wide. Posterior vagina (Fig. 3H, I) elongate. The ventral wall of posterior vagina with two nearly oblong sclerites basally; the dorsal wall with a small long sclerite in the middle aera.

#### Etymology.

The specific name is derived from the Latin word “*aspina*”, referring to the apex of left side of periandrium without process.

#### Host plant.

Bamboo (Poaceae, Bambuseae).

#### Distribution.

China (Hunan).

#### Remarks.

The male genitalia of *K. aspina* sp. nov. is similar to *K. furcata* sp. nov., but diﬀers in: (1) endosoma with one spinous process (endosoma with three spinous processes in *K. furcata*); (2) base of ventral margin of the periandrium without a furcate process (base of ventral margin of periandrium with a long furcate process in *K. furcata*) (3) apical margin of gonostyli round in lateral view (the latter transversal).

### Kirbyana furcata

Taxon classificationAnimaliaHemipteraCixiidae

Zhi & Chen sp. nov.

40321B7C-1521-5C38-AB80-F0206C1C644B

http://zoobank.org/2DD2C8C6-5FF9-490F-AE20-0C70C0FE3CDB

Figures 4 , 5

#### Type material.

***Holotype*:** ♂, China: Yunnan Province, Maguan County, Dulong Town, Jinzhuping Village (22°56'N, 104°30'E), 14 August 2017, leg. Yan Zhi, Qiang Luo and Nian Gong; ***Paratypes***: 1♂1♀, Guangxi Zhuang Autonomous Region, Hechi City, Jinchengjiang Park (24°41'N, 108°3'E), 17 July 2015, leg. Ying-Jian Wang.

#### Description.

Body length: male 4.4‒5.3 mm (*N* = 2), female 5.2 mm (*N* = 1).

**Figure 4.**
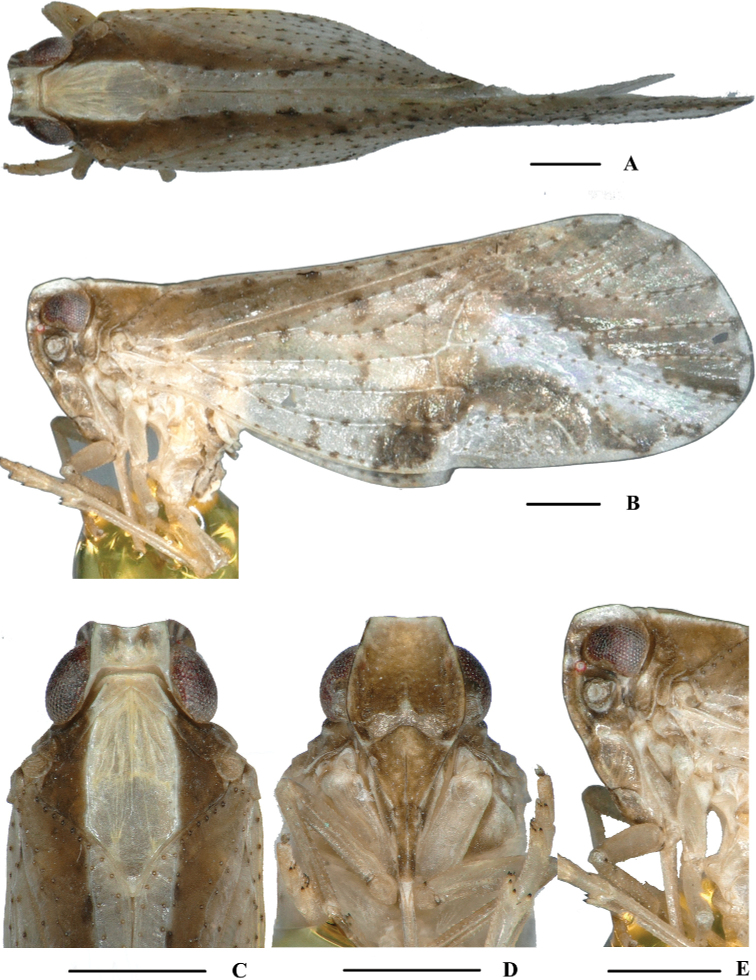
*Kirbyana furcata* sp. nov., male **A** habitus, dorsal view **B** habitus, lateral view **C** head and thorax, dorsal view **D** face, ventral view **E** head, lateral view. Scale bars: 0.5 mm.

***Coloration*.** General color light brown (Fig. 4A–E). Eyes blackish brown, ocelli light yellow, semitransparent. Vertex generally yellowish white. Face generally brown; rostrum light brown. Pronotum with discal areas and mesonotum with area between lateral carinae yellowish white, lateral areas brown. Forewing light brown, semi-translucent, the basal half dotted with small dark brown spots and distal half with two large dark brown patches; small dark brown spots on the ends of longitudinal veins; stigma light brown. Hind tibiae yellowish brown and abdominal sternites dark brown.

**Figure 5.**
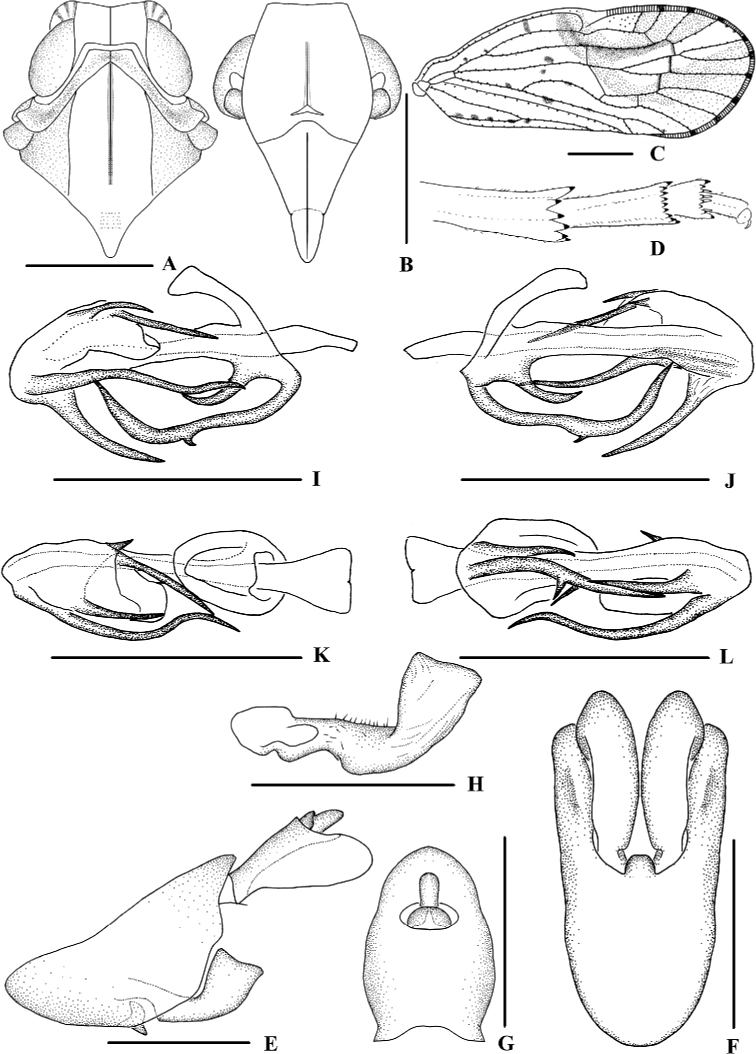
*Kirbyana furcata* sp. nov., male **A** head and thorax, dorsal view **B** face, ventral view **C** forewing **D** apex of left hind leg, ventral view **E** genitalia, lateral view **F** pygofer and gonostyli, ventral view **G** anal segment, dorsal view **H** gonostyli, inner lateral view **I** aedeagus, right side **J** aedeagus, left side **K** aedeagus, dorsal view **L** aedeagus, ventral view. Scale bars: 0.5 mm (**A, B, E–L**); 1.0 mm (**C**).

***Head and thorax*.** Vertex (Figs 4C, 5A) broad, 1.7 times wider than long; anterior margin truncated, posterior margin arched and recessed. Frons (Figs 4D, 5B) widest slightly below the level of antennae, 1.1 times as long as wide; frontoclypeal suture nearly concave into an arch; middle carina with basal half absent; lateral carinae distinct and slight elevated. Pronotum (Figs 4C, 5A) 1.8 times longer than vertex; median carina indistinct, posterior margin nearly at right angle. Mesonotum 1.6 times longer than pronotum and vertex combined. Forewing (Fig. 5C) 2.5 times longer than wide, with 11 apical and 6 subapical cells; fork Sc+RP basad of fork CuA_1_+CuA_2_, first crossvein r-m slightly basad of fork MP, RP two branches, MP with five terminals: MP_11_, MP_12_, MP_2_, MP_3_ and MP_4_, fork MP_1_+MP_2_ basad of fork MP_3_+MP_4_. Metatibiotarsal formula: 6/9/9, second segment of hind tarsus with four platellae (Fig. 5D).

***Male genitalia*.** Pygofer (Fig. 5E, F) symmetrical, dorsal margin concave and U-shaped, slightly widened towards apex and concaved medially in ventral view; in lateral view, lateral lobes trapezoidally extended caudally, medioventral process round in ventral view. Anal segment (Fig. 5E, G) broad, dorsal margin almost straight, apical half of ventral margin convex, apical lobes round in lateral view; 1.5 times longer than wide in dorsal view; anal style strap-shaped, not beyond anal segment. Gonostyli (Fig. 5E, F, H) symmetrical in ventral view; in inner lateral view, base of ventral margin concave, dorsal margin bending inwards at a nearly right angle in the middle, apical part extended, apical margin transversal. Aedeagus (Fig. 5I–L) with total of seven processes. On right side, apex of periandrium with a long spinous process, sinuous, apex directed right-ventrocephalically; base of ventral margin with a long furcate process, one ramus large, apex strongly curved and directed dorsocaudally, the other ramus rather small; a shorter curved spinous process on ventral margin near base, apex directed dorsocaudally; apex of periandrium with a medium-sized spinous process, slightly curved and directed ventrocephalically. Endosoma moderately sclerotised, relatively short, generally curved dorsally. Three spinous processes on or near the apex, the right one medium-sized, slightly curved and directed ventrocephalically; the middle one on the dorsal margin, longest and straight, apex directed right-ventrocephalically; the left one extremely short, apex directed cephalad.

#### Etymology.

The specific name is derived from the Latin word “*furcata*”, referring to the base of ventral margin of periandrium with a long furcate process.

#### Host plant.

Bamboo (Poaceae, Bambuseae).

#### Distribution.

China (Guangxi, Yunnan).

#### Remarks.

This species can be distinguished from the other species of the genus by the following characters: ventral margin of periandrium with three spinous process, two on base and one on apex; apex of periandrium with a long spinous process on the right side; endosoma with three spinous processes on or near the apex.

## Discussion

Prior to this study, nothing had been reported on the host plants of *Kirbyana* except that *K deventeri* (Kirkaldy, 1907) fed on *Saccharum officinarum* L. (Poales, Poaceae) (Kirkaldy 1907). As far as we have observed during our field trips, these two new species, *K. aspina* Zhi & Chen, sp. nov. and *K. furcata* Zhi & Chen, sp. nov. from southern China, were collected on bamboo (Poaceae, Bambuseae), which might be the plant on which they feed.

**Figure 6.**
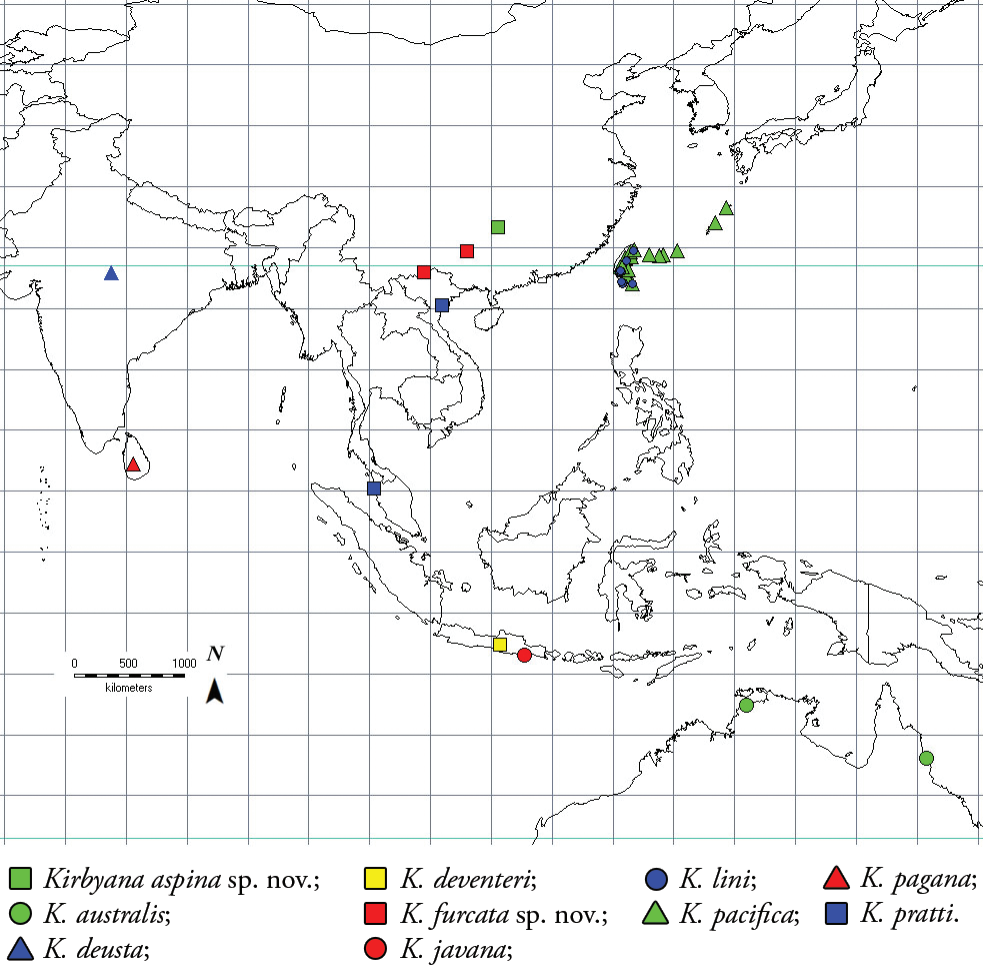
Distribution records of species from the genus *Kirbyana*: *K. aspina* sp. nov. (green square); *K. australis* (green circle); *K deusta* (blue triangle); *K deventeri* (yellow square); *K. furcata* sp. nov. (red square); *K javana* (red circle); *K lini* (blue circle); *K pacifica* (green triangle); *K. pagana* (red triangle); *K pratti* (blue square).

Based on data from published information and our field surveys, the distribution of *Kirbyana* is mostly restricted to the Australian and Oriental regions (Fig. 6) (Holt et al. 2013).

## Supplementary Material

XML Treatment for Kirbyana

XML Treatment for Kirbyana aspina

XML Treatment for Kirbyana furcata

## References

[B1] Bourgoin T (1987) A new interpretation of the homologies of the Hemiptera male genitalia, illustrated by the Tettigometridae (Hemiptera, Fulgoromorpha). In: Proceedings of the 6^th^Auchenorrhyncha Meeting, Turin, Italy, 7–11 September 1987, 113–120.

[B2] Bourgoin T (1993) Female genitalia in HemipteraFulgoromorpha, morphological and phylogenetic data. Annales de la Société Entomologique France 29(3): 225–244.

[B3] Bourgoin T (2021) FLOW (Fulgoromorpha Lists on the Web): a world knowledge base dedicated to Fulgoromorpha. Version 8, updated 18 February 2021. http://hemiptera-databases.org/flow/ [Accessed on: 2021-2-19]

[B4] Bourgoin T Wang RR Asche M Hoch H Soulier-Perkins A Stroiński A Yap S Szwedo J (2015) From micropterism to hyperpterism: recognition strategy and standardized homology-driven terminology of the forewing venation patterns in planthoppers (Hemiptera: Fulgoromorpha). Zoomorphology 134: 63–77. 10.1007/s00435-014-0243-6 25705075 PMC4326643

[B5] Distant WL (1906a) Rhynchota. III. (HeteropteraHomoptera). In: Bingham CT (Ed.) The fauna of British India including Ceylon and Burma. Taylor & Francis, London, 503 pp. 10.1080/00222930608562592

[B6] Distant WL (1906b) Bibliographical and nomenclatorial notes on the Rhynchota. The Entomologist 39: 274–275.

[B7] Distant WL (1911) LXXXV.—Descriptions of new genera and species of Oriental Homoptera. Annals and Magazine of Natural History (8), 48: 735–747. 10.1080/00222931108693092

[B8] Emeljanov AF Hayashi M (2007) New Cixiidae (Hemiptera, Auchenorrhycha) from the Ryukyus, Japan. Japanese Journal of Systematic Entomology 13(1): 127–140.

[B9] Fennah RG (1978) Fulgoroidea (Homoptera) from Vietnam. Annales Zoologici (Warszawa) 34(9): 207–279.

[B10] Fennah RG (1980) The genus *Bajauana* and two allied new genera in New Guinea (Fulgoroidea: Cixiidae). Pacific Insects 22: 237–328.

[B11] Holt BG Lessard JP Borregaard MK Fritz SA Araújo MB Dimitrov D Fabre PH Graham CH Graves GR Jønsson KA Nogués-Bravo D Wang Z Whittaker RJ Fjeldså J Rahbek C (2013) An update of Wallace’s zoogeographic regions of the world. Science 339: 74–78. 10.1126/science.1228282 23258408

[B12] Holzinger WE Emeljanov AF Kammerlander I (2002) The family Cixiidae Spinola, 1839 (Hemiptera: Fulgoromorpha)-a Review. Denisia 4: 113–138.

[B13] Kirkaldy GW (1906) Bibliographical and nomenclatorial notes on the Hemiptera. No. 6. The Entomologist 39: 247–249. 10.5962/bhl.part.1563

[B14] Kirkaldy GW (1907) Descriptions et remarques sur quelques Homoptères de la famille des Fulgoroideae vivant sur la canne à sucre. Annales de la Société entomologique de Belgique. Bruxelles 51: 123–127.

[B15] Löcker B Fletcher MJ Gurr GM (2010) Taxonomic revision of the Australian Eucarpiini (Hemiptera: Fulgoromorpha: Cixiidae) with the description of nine new species. Zootaxa 2425: 1–31. 10.11646/zootaxa.2425.1.1

[B16] Melichar L (1903) Homopteren-Fauna von Ceylon. Verlag von Felix L Dames, Berlin, 248 pp.

[B17] Tsaur SC Hsu TC (2003) The Cixiidae of Taiwan, Part VII: Tribe Pintaliini (Hemiptera: Fulgoroidea). Zoological Studies 42(3): 431–443.

